# Potential Anticancer Properties of Osthol: A Comprehensive Mechanistic Review

**DOI:** 10.3390/nu10010036

**Published:** 2018-01-03

**Authors:** Yalda Shokoohinia, Fataneh Jafari, Zeynab Mohammadi, Leili Bazvandi, Leila Hosseinzadeh, Nicholas Chow, Piyali Bhattacharyya, Mohammad Hosein Farzaei, Ammad Ahmad Farooqi, Seyed Mohammad Nabavi, Mükerrem Betül Yerer, Anupam Bishayee

**Affiliations:** 1 Pharmaceutical Sciences Research Center, School of Pharmacy, Kermanshah University of Medical Sciences, Kermanshah 67146, Iran; yshokoohinia@kums.ac.ir (Y.S.); fataneh.jafari@yahoo.com (F.J.); lhosseinzadeh90@yahoo.com (L.H.); 2 Department of Pharmacognosy and Biotechnology, School of Pharmacy, Kermanshah University of Medical Sciences, Kermanshah 67146, Iran; 3 Students Research Committee, School of Pharmacy, Kermanshah University of Medical Sciences, Kermanshah 67146, Iran; zeynabmohamadi47@yahoo.com (Z.M.); leilibazvandi@yahoo.com (L.B.); 4 Department of Clinical and Administrative Sciences, College of Pharmacy, Larkin University, Miami, FL 33169, USA; NChow@ularkin.org; 5 School of Health Sciences, University of Turabo, Gurabo 00778, Puerto Rico; pbhattacharyya@suagm.edu; 6 Laboratory for Translational Oncology and Personalized Medicine, Rashid Latif Medical College, Lahore 54000, Pakistan; ammadfarooqi@rlmclahore.com; 7 Applied Biotechnology Research Center, Baqiyatallah University of Medical Sciences, Tehran 1435916471, Iran; Nabavi208@gmail.com; 8 Department of Pharmacology, Faculty of Pharmacy, University of Erciyes, 38039 Kayseri, Turkey; eczbetul@yahoo.com; 9 Department of Pharmaceutical Sciences, College of Pharmacy, Larkin University, Miami, FL 33169, USA

**Keywords:** osthol, cancer, phytochemicals, natural product, malignancies, apoptosis

## Abstract

Cancer is caused by uncontrolled cell proliferation which has the potential to occur in different tissues and spread into surrounding and distant tissues. Despite the current advances in the field of anticancer agents, rapidly developing resistance against different chemotherapeutic drugs and significantly higher off-target effects cause millions of deaths every year. Osthol is a natural coumarin isolated from Apiaceaous plants which has demonstrated several pharmacological effects, such as antineoplastic, anti-inflammatory and antioxidant properties. We have attempted to summarize up-to-date information related to pharmacological effects and molecular mechanisms of osthol as a lead compound in managing malignancies. Electronic databases, including PubMed, Cochrane library, ScienceDirect and Scopus were searched for in vitro, in vivo and clinical studies on anticancer effects of osthol. Osthol exerts remarkable anticancer properties by suppressing cancer cell growth and induction of apoptosis. Osthol’s protective and therapeutic effects have been observed in different cancers, including ovarian, cervical, colon and prostate cancers as well as chronic myeloid leukemia, lung adenocarcinoma, glioma, hepatocellular, glioblastoma, renal and invasive mammary carcinoma. A large body of evidence demonstrates that osthol regulates apoptosis, proliferation and invasion in different types of malignant cells which are mediated by multiple signal transduction cascades. In this review, we set spotlights on various pathways which are targeted by osthol in different cancers to inhibit cancer development and progression.

## 1. Introduction

Cancer is a multifaceted and therapeutically challenging disease and rapidly emerging pre-clinical and clinical studies have started to shed light on the molecular mechanisms which underlie cancer development and progression [1]. Based on the United States National Cancer Institute’s categorization, diverse types of cancer include myeloma, carcinoma, leukemia, lymphoma and central nervous system cancer depending on cell type involved [2]. 

In the next couple of decades, the prevalence of cancer is predicted to rise to 70%, amounting to 22 million cases. The most common sites of cancer diagnosed in 2012 were lung, breast, prostate, colon, stomach, cervix and liver carcinomas. One-third of cancer deaths can be attributed to five life style and nutritive factors, such as overweight, low fresh and fiber food intake, absence of physical activity as well as tobacco and alcohol use [1]. Moreover, malignancies affect the psychological well-being (e.g., depression, anxiety, distress and somatization) of patients and their caregivers [3].

The goals of cancer treatment are to destroy tumors or to markedly prolong survival and improve a patient’s quality of life [1]. Deregulations in spatio-temporally controlled signaling mechanisms, including nuclear factor kappa-light-chain-enhancer of activated B cells (NF-κB) [4], activator protein-1 (Ap-1) [4,5] and mitogen-activated protein kinase (MAPK) signaling pathways, played contributory role in carcinogenesis and drug resistance [6,7,8]. Additionally, microtubules have been targeted to disrupt the normal function of the mitotic spindle [9]. Although chemotherapy and radiotherapy are greatly efficient approaches in the treatment of cancer, malignant cells continue to develop resistance to these treatments [4]. 

Natural products, containing bioactive secondary metabolites, have beneficial effects on human health [10,11] and the active ingredients are strong candidates to be lead compounds for the development of new drugs [12]. From 1930 to 2012, 183 drugs were approved as antitumor agents; 30% of these were obtained from natural sources, 57% were natural agents with semisynthetic modifications and 34% had natural product mimetic pharmacophores [13]. Although targeted therapies, such as monoclonal antibodies, have greatly improved, the perfect treatment of several cancers, such as leukemia, gastrointestinal and breast cancers, remain to be achieved through new therapies [10]. Natural products exert anticancer effects through various mechanisms such as alteration of cell cycle [4,14] interference with microtubules [15], topoisomerase inhibitory activities [16], immunomodulatory effects [17] and chemopreventive effects [18] achieved by modulation of various oncogenic signaling molecules and pathways [19,20,21]. 

Coumarins are derivatives of 2H-1-benzopyran-2-one which can be obtained mainly from cyclization of a C-2 oxygenated *cis*-cinnamic acid. These compounds are widely found in plants in the form of free coumarins or their glycosides [22]. Coumarins naturally occurr with several molecular structures, especially in the Apiaceae [23,24] and Rutaceae families, as well as many other plant families, including Asteraceae, Poaceae and Rubiaceae [22]. Coumarins have various biological properties related to their chemical structure [25,26,27]. Some coumarins have vasorelaxant activity in coronary vessels and some other showed hypotensive [22], antiviral [28], antileishmanial [29] anti-inflammatory [30] and antispasmodic [31] effects. Furanocoumarins are also widely used in the treatment of leucoderma and psoriasis due to their photosensitivity properties [32,33]. Some hydroxy- and methoxy-coumarins are able to absorb ultraviolet radiation and are commonly used in sunblock creams [22]. 

Coumarins have prominent anticancer properties with low adverse effects based on the functional groups in the original structure [34,35]. They can affect different cellular pathways, including suppression of angiogenesis, several types of heat shock proteins (HSPs) and cell proliferation as well as inhibition of the enzymes chiefly involved in the pathophysiology of cancer, such as telomerase, monocarboxylate transporters, carbonic anhydrase, aromatase and sulfatase [35,36]. 

Osthol (osthole), 7-methoxy-8-(3-methyl-2-butenyl)-2*H*-1-benzopyran-2-one, a natural coumarin obtained from *Cnidium* spp. and other Apiaceous plants [37], has been found to exert health-promoting effects [38]. *Cnidium monnieri* fruits rich in osthol are popularly used in traditional Chinese medicine [39]. Osthol is a prenylated coumarin with a wide range of pharmacological effects, such as neuroprotective [40], spasmolytic [41] immunomodulatory [42], osteogenic [43], hepatoprotective [44], vasorelaxant [45], antimicrobial [45], antiviral [46] and antileishmanial properties [47], many of which could be implicated in the primary or secondary prevention of cancer. In this review, we aim to provide a critical and mechanistic insight into the biological and pharmacological properties of osthol that are useful in the treatment of cancer.

## 2. Literature Search Methodology

Electronically available databases, including PubMed, Science Direct, Cochrane library and Scopus, were searched for cellular, animal or human studies which assessed the anticancer effects of osthol. We have followed the preferred reporting items for systematic review and meta-analysis (PRISMA) criteria which are preferred for reporting systematic reviews. Relevant and high-quality publications were collected for the years 1966–2017 (up to January). Unpublished results were not included. Only English language papers were included in this review. The search terms were “osthol” or “osthole” in title and abstract. Results from primary search were screened by two independent investigators. Included articles were checked for verification of scientific name of source plant, the dose of administration, type of cancer as well as type of cell line for cellular studies and the animal model for animal studies. Results were reviewed for significant effects on proliferation, apoptosis, pro-inflammatory cytokines, oxidative markers, antioxidant enzymes and tissue damage biomarkers. 

**Figure 1:**
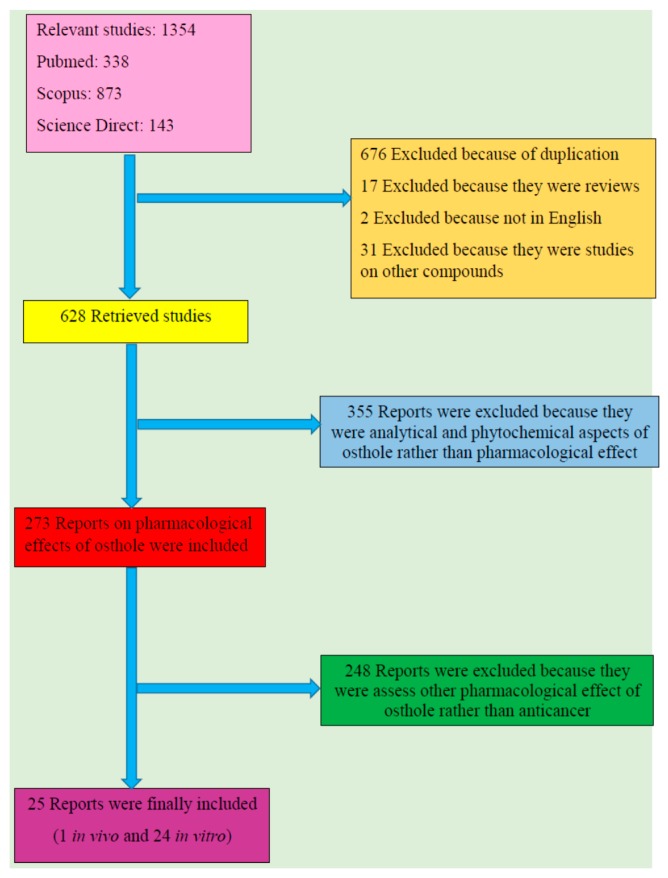
Study selection diagram.

From total 1354 results, 676 reports were excluded because of duplication and 17 were omitted as they were review papers. Additionally, two were ruled out since they were not in English and 31 were excluded because the subject was on other compounds rather than osthol. Among 628 retrieved studies, 355 were excluded out as they were analytical and phytochemical aspects of osthol rather than pharmacological effects; 273 reports on pharmacological effects of osthol were retrieved amongst which 248 were omitted since they assessed pharmacological effects of osthol other than anticancer properties. Twenty five reports including one in vivo and 24 in vitro studies were finally included. Figure 1 shows a flow diagram of the selective procedure for literature included in this review.

## 3. Cellular and Molecular Mechanisms of Anticancer Effects of Osthol

**Figure 2:**
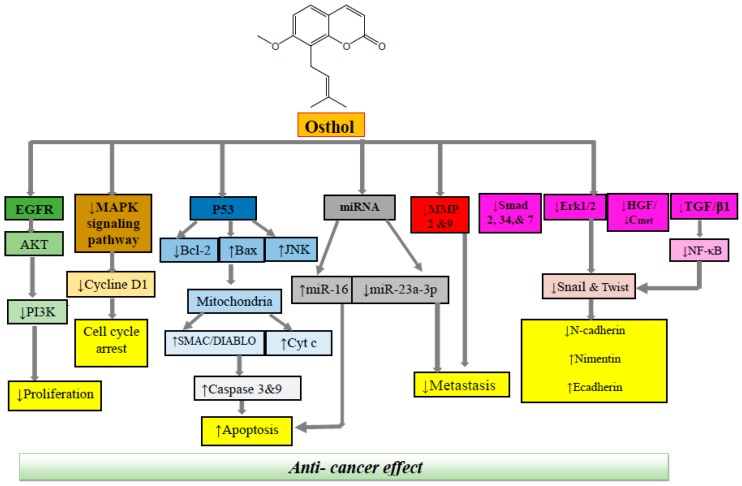
Molecular mechanisms underlying anticancer effect of osthol. EGFR, epidermal growth factor receptor; Akt, AKR mouse thymoma kinase; MAPK, mitogen-activated protein kinase; RNA, ribonucleic acid; MMP, matrix metalloprotease; HGF, hepatocyte growth factor; TGF, tumor growth factor; NF-κB, nuclear factor-κB.

A comprehensive review of the reported literature on anticancer activity of osthol indicated that this therapeutic agent could potentially exert anticancer and antitumor activity via several mechanisms, including cell cycle inhibition, apoptosis induction, anti-angiogenesis, inhibition of metastasis, and suppression of cell proliferation and cell migration (Figure 2). Based on the type of cancer, the effective dose and the mechanism of action could be different. A detailed data on the anticancer mechanism of osthol is presented below and Table 1. 

### 3.1. Colon Cancer

Epidemiological and scientific studies have considerably enhanced our understanding of the evolutionary process underpinning colon cancer development and progression. Metastasis of primary tumors affects the survival of patients [65]. In a study conducted by Huang et al. [48] using HCT116 and SW480 human colon cancer cell lines, osthol demonstrated specific antitumor effect with concentrations of 1, 3 and 10 μM (Table 1). Osthol significantly decreased cell motility in both cell lines through activation of pro-apoptotic signaling pathways and up-regulation of p53 expression; p53 protein has a main role in the regulation of several genes involved in growth inhibition process, apoptosis, cell cycle arrest as well as DNA repair [66]. Apoptosis-induction capacity has been accepted as a mechanism of action for the antitumor drugs [12]; therefore, considerable effort is being directed towards the development of potential medicines that induce apoptosis in tumor cells.

Apoptosis can occur in two main pathways: the mitochondrial (intrinsic) pathway and the death receptor (extrinsic) pathway [67]. In the intrinsic pathway, the mitochondria have a principle role. It is characterized by cytochrome c release from the mitochondrial which activates a family of cysteine protease enzymes, caspases. This process is controlled by the Bcl-2 family of proteins. This family is an essential member of the programmed cell death process, acting to either inhibit (Bcl-2 and Bcl-xl) or promote (Bax and Bcl-xS) cell death [68]. The elevation of the Bax/Bcl-2 ratio, which has a pivotal contribution in apoptosis, is considered as one of the important mechanisms of osthol in the induction of apoptosis and disturbance in permeability of mitochondrial membranes in cancer cells. Osthol exerted apoptotic effect on colon cancer cell line via several mechanisms, including enhancement of the p53 phosphorylation on Ser15 (p-p53) and p53 acetylation on Lys379 (acetylp53). The p53 protein, acting as a tumor suppressor, plays key roles in activating apoptosis through sensing both intrinsic and extrinsic stresses [69]. Moreover, p53 protein has a significant participation in the regulation of cell cycle arrest, and DNA repair, activating c-Jun N-terminal kinase (JNK) pathway, generation of ROS, modulating PI3K/Akt signaling pathway as well as promoting G2/M arrest. Another mechanism which is involved in pro-apoptotic activity of osthol is p53-independent apoptosis process via stimulating JNK and reactive oxygen species (ROS) formation as summarized in Figure 2 [48].

### 3.2. Prostate Cancer

Genome sequencing and gene expression analyses have highlighted essential roles of epigenetic and genetic changes in prostate carcinogenesis. Almost all prostate cancers are adenocarcinomas. Mounting evidences have suggested that prostate cancer is found more often in African-American men in comparison to white men [70]. In a cellular study performed by Shokoohinia et al. [18], potential anticancer effect of osthol was assessed on PC3 human prostate cancer cell line. Results of this investigation suggested that osthol acts as a powerful cytotoxic agent against PC3 cells. Caspase activation through intrinsic or extrinsic pathway was significantly involved in the induction of apoptotic cell death. Osthol could remarkably boost the expression of caspase-9 and caspase-3 in PC3 cells. This natural coumarin also activated apoptosis by down-regulation of the antiapoptotic agent, Bcl-2, and up-regulation of the proapoptotic gene Bax (BCL2-Associated X Protein) as well as Smac/DIABLO, a mitochondrial protein released in response to apoptosis stimuli and suppresses the activity apoptosis inhibitors; thus, can facilitate apoptosis [67]. 

It has previously been convincingly revealed that ectopic expression of miR-23a-3p in DU145 cells induced considerable reversal of osthol-mediated reduction in invasive potential of prostate cancer cells. Detailed mechanistic insights suggested that osthol markedly downregulated miR-23a-3p in DU145 cells [50].

### 3.3. Breast Cancer

Breast cancer is a common malignancy responsible for cancer-related deaths in females around the world, and accordingly, exploring therapeutic approaches in order to suppressing this disease is immediately vital [71]. In an in vitro study reported by Ye et al. [51] on MDA-MB-231 and 4T1, two invasive mammary carcinoma cell lines, osthol at 15 µM showed inhibitory effect on cell proliferation and invasion. Results showed that osthol in combination with platycodin D, a triterpene saponin, could dramatically reduce tumor growth factor-β receptor II (TGFβRII), Smad2, Smad3 and Smad4 gene or protein expressions and efficiently suppressed TGF-β-induced Smad2 and Smad3 phosphorylation. The latter is the main mechanism of osthol in the reduction of proliferation and invasion of breast cancer cells. Hepatocyte growth factor (HGF) is able to induce epithelial–mesenchymal passage in cancerous cells which can result in cancer cell migration. In an in vitro study performed by Hung et al. [48], a series of human breast cancer cells, such as MDA-MB-453, MDA-MB-23, BT-20 and MCF-7, were treated with osthol. It has been observed that osthol significantly suppressed HGF-induced cell distribution, invasion and migration in MCF-7 cell cultures. Abnormal stimulation of the HGF/c-Met (cellular mesenchymal to epithelial transition factor) pathway has a remarkable role in the progression of various models of cancers as well as advancement of tumor invasion and metastatic system. Osthol inhibited HGF-induced c-Met phosphorylation along with a reduction in the total c-Met protein expression in MCF-7 cells which is intervened by C75 (pharmacological inhibitor) of fatty acid synthase (FASN).

In addition to the effects of osthol in breast cancer, this compound reduces the metastasis of this cancer to the bone marrow. In an in vivo study [54], the researchers used a mouse model to investigate the preventive effect of osthol against the metastasis of human breast cancer cells to the bone. Results showed that osthol blocked breast cancer cell growth, migration and invasion, along with enhancement of apoptosis of breast cancer cells. 

Osteoprotegerin (OPG) is a soluble decoy receptor which lacks a trans-membrane domain. It protects the skeleton from excessive bone resorption. Mechanistically, it was shown that OPG interacted with receptor activator of nuclear factor-κB ligand (RANKL) and prevented its structural binding with RANK. The role of osthol in prevention of bone marrow metastasis is mediated by the regulation of OPG/RANKL cascade in the interactions between osteoblasts and breast cancer cells and also suppressing TGF-β/Smads pathway which has a pivotal role in breast cancer bone metastasis. However, many researchers are trying to find strategies to suppress tumor growth as well as tumor metastasis [72].

### 3.4. Brain Cancer

Glioma, a highly relapsing type of tumor, represents 44.6% of central nervous system tumors and has a high rate of morbidity [73]. In vitro investigation by Lin et al. [55] on glioma cell line U87 showed a significant inhibition of proliferation and augmentation of apoptosis at concentrations of 50, 100 and 200 μΜ of osthol. Mechanistically, it has been shown that osthol upregulated miR-16 in the U87 cells. These effects were mediated through up-regulation of expression miR-16 and down-regulation of matrix metalloprotease (MMP)-9 expression [74]. MicroRNAs (miRNAs) are a class of non-coding, small molecule RNAs and act as regulators of gene expression [55]. Ding et al. [56] investigated the anticancer effect of osthol on C6 rat glioma cell. Results showed that osthol apparently prevented glioma cell proliferation. This natural compound was also able to induce apoptosis by up-regulating the expression of pro-apoptotic proteins as well as reduction of anti-apoptotic factors expression. Furthermore, the compound could inhibit C6 cell migration and invasion. Results showed that inhibition occurred through phosphatidylinositol-3-kinase (PI3K)/AKR mouse thymoma kinase (Akt) and MAPK signaling pathway [75]. MAPK pathway was significantly involved in regulation of the phospho-proteome of brain tumors. MAPK activation can also induce cell-cycle arrest via cyclin D1 activation and reduction of apoptosis through modulation of the BCL-2-family [76]. Several proteins control cell proliferation processes, amongst which cyclin-dependent kinases (CDKs) are the most important ones. A CDK binds a regulatory protein called cyclin. This complex (CDK- cyclin) has a modulatory role in cell cycle progression and drive cells to enter the next phase at the appropriate time. Increased cyclin D1 expression has been involved in several types of cancer. Osthol is demonstrated to reduce D1 expression. The PI3K/Akt signaling pathway is involved in regulation of cancer development and progression mainly through triggering an increase in phosphorylated levels of Akt [77].

Glioblastoma multiform (GBM) is a progressive type of brain tumor in adults. Recent treatment approaches for GBM include surgical resection, radiotherapy and chemotherapy [78,79]. An in vitro study on insulin-like growth factor (IGF)-1-induced GBM8401 cells exposed to different concentration of osthol showed that this natural agent can reverse IGF-1-induced morphological changes, mediated by increasing epithelial marker expression and reducing mesenchymal marker expression. It has been found that IGF-1 can result in the conversion of GBM8401 cells to fibroblastic phenotype and in this condition the intercellular space becomes expanded. In addition, a wound-healing experiment indicated that osthol could suppress IGF-1-induced migration of GBM8401 cells. Suppression of MMP-2 and MMP-9 plays a significant role in the inhibition of IGF-1-induced cell migration in GBM8401 cells. Osthol reduced the phosphorylation of Akt and glycogen synthase kinase 3β (GSK3β) and regained the GSK3β activity [57]. Pretreatment with IGF-1 can lead to phosphorylation of Akt and Erk1/2 involved in expression of Snail and Twist. Osthol can remarkably suppress the IGF-1-induced down-regulation of ZO-1 and β-catenin as well as up regulation of vimentin, N-cadherin, Snail and Twist in a dose- and time-dependent manner. Suppressing Snail and Twist expression which is characteristic of mesenchymal tumor areas, is one of the main mechanism of osthol in inhibiting the induction of EMT in epithelial neoplasms [80]. One of the mechanisms by which the growth suppressive effect of osthol suppresses the growth of the malignant cells is mediated via the effects of osthol on the PI3K/Akt mTOR pathway. EMT is the critical step for metastatically competent brain cancer cells to spread and invade distant sites. This process is mediated through growth factors. It is interesting that osthol is able to inhibit IGF-1-induced EMT through PI3K/Akt pathway inhibition in human brain cancer cells [81]. 

### 3.5. Lung Cancer

Lung cancer is the leading cause of cancer-related deaths all over the world, and non-small cell lung cancer (NSCLC) is responsible for about 80% of all cases [82,83]. In an in vitro study by Xu et al. [84], A549 human lung cancer cells were exposed to osthol at various concentrations. Osthol significantly reduced cell growth and arrested the cells in G_2_/M phase. It has been revealed that the osthol cellular mechanism of action includes down-regulation of cyclin B1, p-Cdc2 and Bcl-2 expressions and up-regulation of Bax expression in A549 cells. Osthol could also suppress the PI3K/Akt signaling pathway which might be one of the molecular mechanisms by which the compound exerts anticancer effects. In another study by Xu et al. [58], the effects of various concentration of osthol on the migratory and invasive potential of A549 cells were evaluated. Osthol dose-dependently exerted inhibitory effects on lung cancer cells and effectively suppressed proliferation, migration and invasion of cancerous cells. Cellular mechanisms which are essential for these effects were associated with the inhibition of MMP-2 and MMP-9 expression in the human lung cancer cells which have a significant role in cell invasion and migration. Moreover, Feng et al. [59] investigated the effect of osthol on adenocarcinomic human alveolar basal epithelial cells (A549). The cancerous cells were treated with osthol in 5–20 mM for 48 h. Results showed that osthol extremely suppressed TGF-β1-induced epithelial-to-mesenchymal transition (EMT), adhesion, invasion and migration in A549 which is mediated by adjusting NF-κB and Snail signaling pathways. This molecule can interact with cell adhesion molecules which are involved in angiogenesis. The process of angiogenesis is under regulation of several pro-angiogenic genes as well as growth factors including epidermal growth factor (EGF), vascular endothelial growth factor (VEGF), basic fibroblast growth factor (bFGF), platelet derived growth factors (PDGF), angiopoetin-1 and 2 and MMPs [81]. 

### 3.6. Leukemia

Chronic myeloid leukemia (CML) is a type of hematologic malignancy [85]. Multidrug resistance (MDR) has an important role in CML chemotherapy failure through drug resistance [86]. Wang et al. [60] showed that osthol has a remarkable effect on CML. In this research, K562/ADM cells were treated with osthol for 24 h. The potential of osthol to overcome MDR caused by P-glycoprotein (P-gp) was measured by the CCK-8 assay in the K562/ADM cell line. Results demonstrated that osthol remarkably reduced P-gp expression through suppression of the PI3K/Akt signaling pathway which is associated with modulation of MDR mediated by P-gp in different types of leukemia. The authors concluded that the PI3K/Akt signaling pathway is a key mechanism triggered in the reversal effect of osthol in the MDR. 

Chou et al. [61] evaluated pharmacological properties of osthol against human leukemia via in vivo and in vitro assessments. P-388 D1 murine leukemia cells were intraperitoneally administered into CDF1 female mice (BALB/c female × DBA/2 male). CDF1 mice with leukemia received osthol (30 mg/kg, orally) once a day for a period of nine days. Data clearly suggested that osthol significantly prolonged lifespan of P-388 D1 tumor-bearing mice by more than 37.5% in comparison to solvent-treated animals. Importantly, survival of one mouse of the osthol-treated group was noted to be more than 60 days.

### 3.7. Cervical Cancer

A common type of malignancy in women all over the world is cervical cancer [87]. In an in vitro study that utilized HeLa human cervical cancer cells, osthol concentration- and time-dependently suppressed cell growth. Furthermore, cytotoxic effects were non-significant in coumarin-treated primary cultured normal cervical fibroblasts which indicates its specific pharmacological effects on cancer cells. It has been revealed that osthol performs its anticancer potential on cervical cancer cells by elevating DNA fragmentation as well as activation of poly (ADP-ribose) polymerase (PARP) which has an essential contribution in programmed cell death resulting in induction of apoptosis in HeLa cells [61]. 

### 3.8. Ovarian Cancer

Ovarian cancer is considered as the most lethal gynecologic cancer [88] in women. Epidemiological studies demonstrated that the proportion of ovarian cancer patients who experienced a five-year survival rate is less than 50% post-diagnosis. The main therapeutic approach for ovarian cancer patients is cytoreductive surgery along with paclitaxel-based chemical agents [88,89]. Clinical evidence reported good primary response in many cases; however, there are still remarkable challenges such as multi-chemotherapy drug resistance. In an in vitro study reported by Jiang et al. [62] uncontrolled proliferation and migration of ovarian cancer cells, including OV2008 and A2780, were assessed. Osthol remarkably reduced cell viability of ovarian cancer cells; whereas no toxicity was detected in normal ovarian cells. Subsequent to treatment with osthol, migratory potential, expression levels and functionalities of MMP-9 and MMP-2 were noted to be significantly suppressed in wound healing and trans-well assays. This natural coumarin repressed cells proliferation via promoting G_2_/M phase cell cycle arrest and activation of apoptosis process in malignant cells. It has been suggested that other underlying mechanisms of its anticancer action were the enhancement of the apoptotic protein caspase-3, caspase-9 and Bax/Bcl-2.

### 3.9. Renal Cancer

In an in vitro study, Min et al. [63] showed that osthol increased TNF-related apoptosis-inducing ligand (TRAIL)-mediated cell death in Caki cell line. Induction of apoptotic cell death by osthol (20–30 µM for 24 h) is mediated by regulation of the FLICE like inhibitory protein (c-FLIP) expression in human renal carcinoma cells. c-FLIP overexpression markedly inhibited apoptosis; however, osthol significantly reduced c-FILP levels and sensitized resistant cells to TRAIL. Also, osthol significantly decreased MMP levels and synergistic treatment with TRAIL induced an increase in cytosolic accumulation of cytochrome c. These findings provided evidence that osthol worked synergistically with TRAIL and induced apoptosis in TRAIL-resistant cell lines. Moreover, osthol blocks the growth and invasion of bladder cancer cells by inhibiting the expression of the angiogenesis-related proteins COX-2, VEGF, MMP-2 and NF-κB. Future studies must converge on detailed investigation of osthol-mediated regulation of the TRAIL pathway in resistant cancer cells. We still have insufficient information about regulation of death receptors (DR4 and DR5) by osthol in cancer cells.

### 3.10. Liver Cancer

In an in vitro study, Lin et al. [64] reported that osthol reduced hepatocellular carcinoma (HCC) cell proliferation. The chemotherapeutic potential of osthol on HCC cell proliferation was mediated through induction of DNA damage and cell cycle arrest as well as inhibition of migration of HCC cells. It remarkably suppressed the cell cycle in the G_2_/M phase by blocking the expression of Cdc2. Cyclin-dependent kinase 1 (CDK1)-cyclin B, also known as cell division control protein kinase 2 (Cdc2)-cyclin B is a member of cyclin-dependent kinases with a significant role in regulation of the cell cycle. It has been found that down-regulation of MMP-2 and MMP-9 is involved in suppression of migration of HCC cells by this natural agent. Finally, the authors demonstrated that osthole inhibited EMT by increasing epithelial biomarkers E-cadherin and β-catenin and simultaneously repressing the levels of N-cadherin and vimentin. This phytoconstituents also damaged DNA by induction of DNA excision repair protein (ERCC) 1 expression enhancing epithelial biomarkers E-cadherin and β-catenin, and reducing mesenchymal N-cadherin are among the main cellular factors which have a key role in suppression of EMT by osthol [90]. These cellular pathways suggest an interesting chemotherapeutic effect of osthol on HCC. The interaction of DNA and other coumarins have been reported previously [91]. 

### 3.11. Protectice Effect against Toxicity of Chemotherpy 

The positive effects of osthol in modulating cancer include direct anticarcinogenic activity along with protective effects against side effects of conventional chemotherapeutic agents. One of the chemotherapeutic drugs that are used for treatment of several types of cancers is doxorubicin [55] which shows several adverse effects due to its inherent pro-oxidant activity. Protective effects of osthol against doxorubicin-induced oxidative stress and apoptosis in the neuronal cell line (PC12) has been confirmed. The protective mechanisms of osthol include enhancement of mitochondrial membrane potential, elevation of Bax/Bcl-2 ratio, improvement in loss of cell viability, suppression of intracellular reactive oxygen species (ROS) generation as well as increases in mitochondrial membrane potential in PC12 cells [18,92]. Fatty acid synthase (FASN) is the only enzyme engaged in long-chain saturated fatty acid synthesis and is implicated in cancer progression by regulating lipid raft function. Thus, one of the molecular pathways by which osthol performs its protective action against cancer progression is its potent inhibitory effect on FASN [53]. 

## 4. Toxicity of Osthol

Reviewing current literature can help to collect information regarding the bio-efficacy and safety of the administration of significant phytochemicals in the prevention and management of different malignancies and their relevant complications. Since osthol safety has been investigated, the no-observed adverse-effect level (NOAEL) of osthol for both male and female rats is considered to be less than 5 mg/kg [93]. We suggest to perform randomized, controlled, trials with adequate sample size in order to validate the safety and efficacy of osthol in managing patients with malignancies.

## 5. Conclusions and Future Directions

Osthol is a natural coumarin isolated from Umbelliferae plants with a wide range of pharmacological effects. The goal of the present review was to provide a summary of current knowledge on the anticancer effects of osthol as a lead compound in malignancy therapy along with in-depth molecular mechanisms. This natural phytochemical suppresses the activation of different apoptotic proteins such as caspase-3 and caspase-9, Smac/DIABLO, poly-ADP ribose polymerase and survivin, which are associated with the intrinsic pathways of apoptosis. Osthol is demonstrated to induce apoptosis in different carcinoma cell lines through up-regulation of p53 expression. It has also been shown that osthol induces apoptosis through a mechanism independent of the tumor-suppressing properties of p53. This effect of osthol seems to be promising since the compound demonstrated antitumor effects in several types of cancers in which a p53 regulatory system is involved [94]. It also can diminish metastasis via different molecular mechanisms such as reducing the expression of Smad 2, 3 and 4, C75, and inhibition of the HGF/c-Met signaling pathway as well as HGF-induced c-Met phosphorylation. Osthol also has a stimulatory effect on the extrinsic apoptotic pathway by increasing levels of caspase-8 (unique to the extrinsic pathway). Furthermore, ability of osthol to inhibit NF-κB-mediated cell survival pathway plays an important contribution in its pro-apoptotic properties, as summarized in Figure 2. 

Results obtained from studies evaluating anticancer potential of osthol have confirmed its protective and therapeutic effect on various types of malignancies including ovarian cancer, cervical cancer, chronic myeloid leukemia, lung adenocarcinoma cells, glioma as well as glioblastoma multiform cells, invasive mammary carcinoma cells, colon cancer, and prostate cancer. Recent investigations, mentioned previously, have suggested that osthol has a significant action in the brain by protecting neurons. Additionally, the ability of osthol in penetrating the blood–brain barrier indicates its potential as a future drug for chemotherapy of brain tumors.

Based on the insights gleaned from decades of research, it seems clear that a “one size fits all” approach will not be effective in clinical settings. There has been a paradigm shift in our understanding about the heterogeneous nature of cancer, and accordingly, researchers are now focusing on multi-targeted approaches. Osthol has emerged as a promising phytochemical reportedly involved in the regulation of different signaling pathways. However, we have just started to scratch the surface of information related to the potential of osthol to target multiple proteins and signaling pathways. We still need to have a better understanding of how osthol regulates specific signaling pathways, such as VEGF/VEGFR, PDGF/PDGFR and SHH/GLI pathways, and exert differential modulation of oncogenic and tumor suppressor microRNAs. 

Numerous studies presented here clearly suggest that osthol possesses the potential to act in an inhibitory role in the progression of malignancies. A large body of evidence demonstrated that osthol regulates apoptosis, proliferation and invasion in different types of malignant cells which is mediated by multiple cellular signaling pathways. However, the mechanisms of function of osthol toward various cancers are not the same. As it was mentioned, osthol possesses antioxidant activity, causing inhibition of ROS overproduction in different cells. ROS can activate several pathways involved in metastasis and possess critical roles in invasion and invadopodia formation; moreover, ROS also possess prominent roles on signaling cascades relating to resistance to apoptosis, neovascularization, and proliferation. Therefore, ROS contribute in various mechanisms [95,96,97,98], and reduction of ROS using antioxidants is a promising way to inhibit different cancers. Based on the different roles of ROS in cancer cells and their contribution in different mechanistic pathways, the function of antioxidants such as osthol in excretion of anticancer activity could be probably different. Regarding the remarkable pharmacological functions of osthol in the protection and treatment of malignancies as described above, this natural compound is becoming a significant natural structure for drug discovery. Furthermore, mechanistic investigations for exploring precise intracellular mechanisms of osthol in defending and fighting against cancer are recommended. Also, well-designed randomized clinical trials are important to evaluate the safety and efficacy of osthol in patients with different types of cancers.

## Figures and Tables

**Table 1:** Pharmacological mechanisms of osthol involved in its anticancer activities.

Type of Cancer	Conc. or Dose	Cancer Model Used	Anticancer Effects and Mechanisms	Reference
Colon	1, 3 & 10 mM	In vitro (HCT116 & SW480 cells)	↓Cell motility; ↑apoptosis; ↑phosphorylation of p53 on Ser15 (p-p53); ↑acetylation of p53; ↑ROS; ↑JNK	[48]
Prostate	100 mM	In vitro (PC3 cells)	↑Apoptosis; ↓Bcl-; ↑Bax; ↑Smac/DIABLO	[49]
Prostate	20~80 μM	In vitro (AIPC, DU145 & PC3 cells)	↓TGF-β, ↓Akt, JNK& ERK ↓miR-23a-3p	[50]
Breast	15 mM	In vitro (MDA-MB-231 & 4T1)	↓TbetaRII; ↓Smad2; ↓Smad3; ↓Smad4	[51]
Breast	20 mM	In vitro (MCF-7, MDA-MB-453, MDA-MB-231 & BT-20 cells)	↓c-Met signaling; ↓FASN; ↓HGF- induced EMT; ↓c-Met protein levels; ↓cell migration; ↓invasion; ↓c-Met/Akt/mTOR	[52]
Breast	5, 10, 24, 40 & 80 mM	In vitro (MDA-MB-231, MCF-7, HBL-100 & HER2-overexpressing human cancer cell lines)	↓proliferation; ↑apoptosis; ↓FASN; ↓Akt; ↓mTOR; ↑paclitaxel-induced cytotoxicity	[53]
Breast	5.25 mg/kg	In vivo (Mice treated orally twice weekly)	↑IL-8; ↑M-CSF; ↑PTHrP; ↓OPG/RANKL	[54]
Breast	20–90 mM	In vitro (MDA-231BO cells)	↓Cell viability; ↓proliferation; ↑apoptosis; ↓TGF-β/Smads	[54]
Brain	50, 100 & 200 mM	In vitro (U87 cells)	↓proliferation; ↑apoptosis; ↑miR16; ↓MMP9	[55]
Brain	25, 50 & 100 mM	In vitro (Rat glioma cells)	↓Proliferation; ↓PI3K/Akt/MAPK	[56]
Brain	10–100 mM	In vitro (GBM8401 cells)	↓EMT; ↓Akt and GSK3β; ↓Snail; ↓Twist; ↓I3K/Akt	[57]
Brain	100 mM	In vitro (SKNMC cells)	↑Apoptosis by ↑Bcl; ↑Bax; ↑Smac/DIABLO	[49]
Lung	50, 100 & 150 mM	In vitro (A549 cells)	↑G2/M arrest; ↑apoptosis; ↓Cyclin B1; ↓p-Cdc2; ↓Bcl-2; ↑Bax, ↓PI3K/Akt signaling pathway	[58]
Lung	20, 40, 60 mM 80 mM	In vitro (A549 cells)	↓MMP-2; ↓MMP-9	[58]
Lung	5–20 mM	In vitro (A549 cells)	↓NF-κB mediated snail activation; ↓invasion; ↓migration; ↓adhesion	[59]
Lung	100 mM	In vitro (H1299 cells)	↑Apoptosis; ↓Bcl; ↑Bax; ↑Smac/DIABLO	[49]
Leukemia	5 mM 15 mM	In vitro (K562/ADM cells)	↓MDR in myelogenous leukemia	[60]
Leukemia	30 mg/kg for 8 days	In vivo (CDF1 female mice transplanted with P-388 D1 cells)	↑Apoptosis; ↓P-388 D1 cells	[61]
Cervix	77.96 mM 64.94 mM	In vitro (HeLa cells)	↑Apoptosis	[61]
Ovary	20, 40, 80, 120, 160 and 200	In vitro (A2780 & OV2008 cells)	↓Cells proliferation; ↑apoptosis	[62]
Ovary	5, 10, 24, 40 mM 80 mM	In vitro (SKOV3 human cancer cells)	↓FASN; ↓proliferation; ↑apoptosis; ↓Akt; ↓mTOR; ↑paclitaxel-induced cytotoxicity	[53]
Renal	20–30 mM	In vitro (Caki & U251MG cells)	↑Apoptosis; ↓MMP level; ↑cytochrome *c*; ↓c-FLIP	[63]
Liver	20, 40, 80, 120, 160 or 200 mM	In vitro (SMCC-7721, MHCC-97H, HCC-LM3 & BEL-7402 cells)	↓Proliferation; ↑DNA damage; ↓migration; ↓Cdc2; ↓cyclin B1; ↑ERCC1	[64]

Arrows (↑ and ↓) show increase and decrease in the obtained variables, respectively. H1299, human non-small cell lung carcinoma; GSK3β, glycogen synthase kinase 3-β; EMT, epithelial-mesenchymal transition; Akt, AKR mouse thymoma kinase; PI3K, phosphatidylinositol-3-kinase; IGF, insulin-like growth factor; EGFR, epidermal growth factor receptor; COX-2, cyclooxygenase 2; TPK, tyrosine protein kinase; VEGF, vascular endothelial growth factor; NF-κB, nuclear factor kappa-light-chain-enhancer of activated B cells; MAPK, mitogen-activated protein kinase; ROS, reactive oxygen species; JNK, c-Jun N-terminal kinase; c-MET, cellular mesenchymal to epithelial transition factor; mTOR, mammalian target of rapamycin; MDR, multiple drug resistance; Bax: BCL2-Associated X Protein; TGFβRII, transforming growth factor-β receptor, type II; GMP, guanosine monophosphate.

## References

[1] World-Health-Organization (2015). Cancer: Fact Sheet No. 297. http://www.who.int.

[2] Jena J., Ranjan R., Ranjan P., Sarangi M.K. (2012). A Study on Natural Anticancer Plants. Int. J. Pharm. Chem. Sci..

[3] Padmaja G., Vanlalhruaii C., Rana S., Nandinee D., Hariharan M. (2016). Care givers’ depression, anxiety, distress, and somatization as predictors of identical symptoms in cancer patients. J. Cancer Res. Ther..

[4] Safarzadeh E., Sandoghchian S.S., Baradaran B. (2014). Herbal medicine as inducers of apoptosis in cancer treatment. Adv. Pharm. Bull..

[5] HemaIswarya S., Doble M. (2006). Potential synergism of natural products in the treatment of cancer. Phytother. Res..

[6] Cragg G.M., Newman D.J. (2005). Plants as a source of anti-cancer agents. J. Ethnopharmacol..

[7] Guan X., Sun Z., Chen X., Wu H., Zhang X. (2012). Inhibitory effects of Zengshengping fractions on DMBA-induced buccal pouch carcinogenesis in hamsters. Chin. Med. J..

[8] Sadeghi-Aliabadi H., Aliasgharluo M., Fattahi A., Mirian M., Ghanadian M. (2013). In vitro cytotoxic evaluation of some synthesized COX-2 inhibitor derivatives against a panel of human cancer cell lines. Res. Pharm. Sci..

[9] Mukhtar E., Adhami V.M., Mukhtar H. (2014). Targeting microtubules by natural agents for cancer therapy. Mol. Cancer Ther..

[10] Basmadjian C., Zhao Q., Bentouhami E., Djehal A., Nebigil C.G., Johnson R.A., Serova M., De Gramont A., Faivre S., Raymond E. (2014). Cancer wars: Natural products strike back. Front. Chem..

[11] Vergara D., De Domenico S., Tinelli A., Stanca E., Del Mercato L.L., Giudetti A.M., Simeone P., Guazzelli N., Lessi M., Manzini C. (2017). Anticancer effects of novel resveratrol analogues on human ovarian cancer cells. Mol. BioSyst..

[12] Ahmadi F., Derakhshandeh K., Jalalizadeh A., Mostafaie A., Hosseinzadeh L. (2015). Encapsulation in PLGA-PEG enhances 9-nitro-camptothecin cytotoxicity to human ovarian carcinoma cell line through apoptosis pathway. Res. Pharm. Sci..

[13] Newman D.J., Giddings L.-A. (2014). Natural products as leads to antitumor drugs. Phytochem. Rev..

[14] Farzaei M.H., Bahramsoltani R., Rahimi R. (2016). Phytochemicals as adjunctive with conventional anticancer therapies. Curr. Pharm. Des..

[15] Nobili S., Lippi D., Witort E., Donnini M., Bausi L., Mini E., Capaccioli S. (2009). Natural compounds for cancer treatment and prevention. Pharmacol. Res..

[16] Ashley R.E., Osheroff N. (2014). Natural products as topoisomerase II poisons: Effects of thymoquinone on DNA cleavage mediated by human topoisomerase IIα. Chem. Res. Toxicol..

[17] Schafer G., Kaschula C.H. (2014). The immunomodulation and anti-inflammatory effects of garlic organosulfur compounds in cancer chemoprevention. Anti-Cancer Agents Med. Chem..

[18] Shokoohinia Y., Hosseinzadeh L., Moieni-Arya M., Mostafaie A., Mohammadi-Motlagh H.-R. (2014). Osthole attenuates doxorubicin-induced apoptosis in PC12 cells through inhibition of mitochondrial dysfunction and ROS production. BioMed Res. Int..

[19] Bishayee A., Sethi G. (2016). Bioactive natural products in cancer prevention and therapy: Progress and promise. Semin. Cancer Biol..

[20] Block K.I., Gyllenhaal C., Lowe L., Amedei A., Amin A.R., Amin A., Aquilano K., Arbiser J., Arreola A., Arzumanyan A. (2015). Designing a broad-spectrum integrative approach for cancer prevention and treatment. Semin. Cancer Biol..

[21] Shanmugam M.K., Lee J.H., Chai E.Z.P., Kanchi M.M., Kar S., Arfuso F., Dharmarajan A., Kumar A.P., Ramar P.S., Looi C.Y. (2016). Cancer prevention and therapy through the modulation of transcription factors by bioactive natural compounds. Semin. Cancer Biol..

[22] Waksmundzka-Hajnos M., Sherma J., Kowalska T. (2008). Thin Layer Chromatography in Phytochemistry.

[23] Sajjadi S., Zeinvand H., Shokoohinia Y. (2009). Isolation and identification of osthol from the fruits and essential oil composition of the leaves of *Prangos asperula* Boiss. Res. Pharm. Sci..

[24] Sajjadi S.E., Shokoohinia Y., Hemmati S. (2012). Isolation and identification of furanocoumarins and a phenylpropanoid from the acetone extract and identification of volatile constituents from the essential oil of *Peucedanum pastinacifolium*. Chem. Nat. Compd..

[25] Ahmadi F., Valadbeigi S., Sajjadi S., Shokoohinia Y., Azizian H., Taheripak G. (2016). Grandivittin as a natural minor groove binder extracted from Ferulago macrocarpa to ct-DNA, experimental and in silico analysis. Chem. Biol. Interact..

[26] Venugopala K.-N., Rashmi V., Odhav B. (2013). Review on natural coumarin lead compounds for their pharmacological activity. BioMed Res Int..

[27] Shokoohinia Y., Gheibi S., Kiani A., Sadrjavadi K., Nowroozi A., Shahlaei M. (2015). Multi-spectroscopic and molecular modeling investigation of the interactions between prantschimgin and matrix metalloproteinase 9 (MMP9). Luminescence.

[28] Ghannadi A., Fattahian K., Shokoohinia Y., Behbahani M., Shahnoush A. (2014). Anti-viral evaluation of sesquiterpene coumarins from *Ferula assa-foetida* against HSV-1. Iran. J. Pharm. Res..

[29] Sajjadi S.E., Eskandarian A.-A., Shokoohinia Y., Yousefi H.-A., Mansourian M., Asgarian-Nasab H., Mohseni N. (2016). Antileishmanial activity of prenylated coumarins isolated from *Ferulago angulata* and *Prangos asperula*. Res. Pharm. Sci..

[30] Kiani A., Almasi K., Shokoohinia Y., Sadrjavadi K., Nowroozi A., Shahlaei M. (2015). Combined spectroscopy and molecular modeling studies on the binding of galbanic acid and MMP9. Int. J. Biol. Macromol..

[31] Sadraei H., Shokoohinia Y., Sajjadi S.E., Mozafari M. (2013). Antispasmodic effects of *Prangos ferulacea* acetone extract and its main component osthole on ileum contraction. Res. Pharm. Sci..

[32] Ceska O., Chaudhary S., Warrington P., Ashwood-Smith M. (1986). Photoactive furocoumarins in fruits of some umbellifers. Phytochemistry.

[33] Ranjbar S., Shokoohinia Y., Ghobadi S., Bijari N., Gholamzadeh S., Moradi N., Ashrafi-Kooshk M.R., Aghaei A., Khodarahmi R. (2013). Studies of the interaction between isoimperatorin and human serum albumin by multispectroscopic method: Identification of possible binding site of the compound using esterase activity of the protein. Sci. World J..

[34] Bijari N., Shokoohinia Y., Ashrafi-Kooshk M.R., Ranjbar S., Parvaneh S., Moieni-Arya M., Khodarahmi R. (2013). Spectroscopic study of interaction between osthole and human serum albumin: Identification of possible binding site of the compound. J. Lumin..

[35] Thakur A., Singla R., Jaitak V. (2015). Coumarins as anticancer agents: A review on synthetic strategies, mechanism of action and SAR studies. Eur. J. Med. Chem..

[36] Geisler J., Sasano H., Chen S., Purohit A. (2011). Steroid sulfatase inhibitors: Promising new tools for breast cancer therapy?. J. Steroid Biochem. Mol. Biol..

[37] Jelodarian Z., Shokoohinia Y., Rashidi M., Ghiasvand N., Hosseinzadeh L., Iranshahi M. (2017). New polyacetylenes from *Echinophora cinerea* (Boiss.) Hedge et Lamond. Nat. Prod. Res..

[38] You L., Feng S., An R., Wang X. (2009). Osthole: A promising lead compound for drug discovery from a traditional Chinese medicine (TCM). Nat. Prod. Commun..

[39] Zhang Q., Qin L., He W., Van Puyvelde L., Maes D., Adams A., Zheng H., De Kimpe N. (2007). Coumarins from *Cnidium monnieri* and their antiosteoporotic activity. Planta Med..

[40] Wang S.-J., Lin T.-Y., Lu C.-W., Huang W.-J. (2008). Osthole and imperatorin, the active constituents of *Cnidium monnieri* (L.) Cusson, facilitate glutamate release from rat hippocampal nerve terminals. Neurochem. Int..

[41] Sadraei H., Shokoohinia Y., Sajjadi S., Ghadirian B. (2012). Antispasmodic effect of osthole and *Prangos ferulacea* extract on rat uterus smooth muscle motility. Res. Pharm. Sci..

[42] Resch M., Steigel A., Chen Z.-L., Bauer R. (1998). 5-Lipoxygenase and cyclooxygenase-1 inhibitory active compounds from *Atractylodes lancea*. J. Nat. Prod..

[43] Zhang W., Ma D., Zhao Q., Ishida T. (2010). The effect of the major components of *Fructus Cnidii* on osteoblasts in vitro. J. Acupunct. Meridian Stud..

[44] Huang R., Chen C., Huang Y., Hsieh D., Hu C., Chang C. (1996). Osthole increases glycosylation of hepatitis B surface antigen and suppresses the secretion of hepatitis B virus in vitro. Hepatology.

[45] Guh J.-H., Yu S.-M., Ko F.-N., Wu T.-S., Teng C.-M. (1996). Antiproliferative effect in rat vascular smooth muscle cells by osthole, isolated from *Angelica pubescens*. Eur. J. Pharmacol..

[46] Gholamzadeh S., Behbahani M., Fattahi A., Sajjadi S., Shokoohinia Y. (2012). Antiviral evaluation of coumarins from *Prangos ferulacea* L. (Lindl). Res. Pharm. Sci..

[47] Kermani E.-K., Sajjadi S.-E., Hejazi S.-H., Arjmand R., Saberi S., Eskandarian A.-A. (2016). Anti-Leishmania activity of osthole. Pharmacog. Res..

[48] Huang S.-M., Tsai C.-F., Chen D.-R., Wang M.-Y., Yeh W.-L. (2014). p53 is a key regulator for osthole-triggered cancer pathogenesis. BioMed Res. Int..

[49] Shokoohinia Y., Hosseinzadeh L., Alipour M., Mostafaie A., Mohammadi-Motlagh H.-R. (2014). Comparative evaluation of cytotoxic and apoptogenic effects of several coumarins on human cancer cell lines: Osthole induces apoptosis in p53-deficient H1299 cells. Adv. Pharmacol. Sci..

[50] Wen Y.-C., Lee W.-J., Tan P., Yang S.-F., Hsiao M., Lee L.-M., Chien M.-H. (2015). By inhibiting snail signaling and miR-23a-3p, osthole suppresses the EMT-mediated metastatic ability in prostate cancer. Oncotarget.

[51] Ye Y., Han X., Guo B., Sun Z., Liu S. (2013). Combination treatment with platycodin D and osthole inhibits cell proliferation and invasion in mammary carcinoma cell lines. Environ. Toxicol. Pharmacol..

[52] Hung C.-M., Kuo D.-H., Chou C.-H., Su Y.-C., Ho C.-T., Way T.-D. (2011). Osthole suppresses hepatocyte growth factor (HGF)-induced epithelial-mesenchymal transition via repression of the c-Met/Akt/mTOR pathway in human breast cancer cells. J. Agric. Food Chem..

[53] Lin V.C.-H., Chou C.-H., Lin Y.-C., Lin J.-N., Yu C.-C., Tang C.-H., Lin H.-Y., Way T.-D. (2010). Osthole suppresses fatty acid synthase expression in HER2-overexpressing breast cancer cells through modulating Akt/mTOR pathway. J. Agric. Food Chem..

[54] Wu C., Sun Z., Guo B., Ye Y., Han X., Qin Y., Liu S. (2017). Osthole inhibits bone metastasis of breast cancer. Oncotarget.

[55] Lin K., Gao Z., Shang B., Sui S., Fu Q. (2015). Osthole suppresses the proliferation and accelerates the apoptosis of human glioma cells via the upregulation of microRNA-16 and downregulation of MMP-9. Mol. Med. Rep..

[56] Ding D., Wei S., Song Y., Li L., Du G., Zhan H., Cao Y. (2013). Osthole exhibits anti-cancer property in rat glioma cells through inhibiting PI3K/Akt and MAPK signaling pathways. Cell. Physiol. Biochem..

[57] Lin Y.-C., Lin J.-C., Hung C.-M., Chen Y., Liu L.-C., Chang T.-C., Kao J.-Y., Ho C.-T., Way T.-D. (2014). Osthole inhibits insulin-like growth factor-1-induced epithelial to mesenchymal transition via the inhibition of PI3K/Akt signaling pathway in human brain cancer cells. J. Agric. Food Chem..

[58] Xu X.-M., Zhang Y., Qu D., Feng X.-W., Chen Y., Zhao L. (2012). Osthole suppresses migration and invasion of A549 human lung cancer cells through inhibition of matrix metalloproteinase-2 and matrix metallopeptidase-9 in vitro. Mol. Med. Rep..

[59] Feng H., Lu J.-J., Wang Y., Pei L., Chen X. (2017). Osthole inhibited TGF β-induced epithelial–mesenchymal transition (EMT) by suppressing NF-κB mediated Snail activation in lung cancer A549 cells. Cell Adhes. Migr..

[60] Wang H., Jia X.-H., Chen J.-R., Wang J.-Y., Li Y.-J. (2016). Osthole shows the potential to overcome P-glycoprotein-mediated multidrug resistance in human myelogenous leukemia K562/ADM cells by inhibiting the PI3K/Akt signaling pathway. Oncol. Rep..

[61] Chou S.Y., Hsu C.S., Wang K.T., Wang M.C., Wang C.C. (2007). Antitumor effects of Osthol from *Cnidium monnieri*: An in vitro and in vivo study. Phytother. Res..

[62] Jiang G., Liu J., Ren B., Tang Y., Owusu L., Li M., Zhang J., Liu L., Li W. (2016). Anti-tumor effects of osthole on ovarian cancer cells in vitro. J. Ethnopharmacol..

[63] Min K.-J., Han M., Kim S., Park J.-W., Kwon T.K. (2017). Osthole enhances TRAIL-mediated apoptosis through downregulation of c-FLIP expression in renal carcinoma Caki cells. Oncol. Rep..

[64] Lin Z.-K., Liu J., Jiang G.-Q., Tan G., Gong P., Luo H.-F., Li H.-M., Du J., Ning Z., Xin Y. (2017). Osthole inhibits the tumorigenesis of hepatocellular carcinoma cells. Oncol. Rep..

[65] Friend S. (1994). p53: A glimpse at the puppet behind the shadow play. Science.

[66] Livingstone L.R., White A., Sprouse J., Livanos E., Jacks T., Tlsty T.D. (1992). Altered cell cycle arrest and gene amplification potential accompany loss of wild-type p53. Cell.

[67] Hosseinzadeh L., Behravan J., Mosaffa F., Bahrami G., Bahrami A.R., Karimi G. (2011). Effect of curcumin on doxorubicin-induced cytotoxicity in H9c2 cardiomyoblast cells. Iran. J. Basic Med. Sci..

[68] Elmore S. (2007). Apoptosis: A review of programmed cell death. Toxicol. Pathol..

[69] Amaral J.D., Xavier J.M., Steer C.J., Rodrigues C.M. (2010). The role of p53 in apoptosis. Discov. Med..

[70] Mao H.L., Liu P.S., Zheng J.F., hai Zhang P., Zhou L.G., Xin G., Liu C. (2007). Transfection of Smac/DIABLO sensitizes drug-resistant tumor cells to TRAIL or paclitaxel-induced apoptosis in vitro. Pharmacol. Res..

[71] Jemal A., Bray F., Center M.M., Ferlay J., Ward E., Forman D. (2011). Global cancer statistics. CA Cancer J. Clin..

[72] De Cicco P., Panza E., Armogida C., Ercolano G., Taglialatela-Scafati O., Shokoohinia Y., Camerlingo R., Pirozzi G., Calderone V., Cirino G. (2017). The hydrogen sulfide releasing molecule acetyl deacylasadisulfide inhibits metastatic melanoma. Front. Pharmacol..

[73] Sun Y.-C., Wang J., Guo C.-C., Sai K., Wang J., Chen F.-R., Yang Q.-Y., Chen Y.-S., Wang J., To T.S.-S. (2014). MiR-181b sensitizes glioma cells to teniposide by targeting MDM2. BMC Cancer.

[74] Gheibi S., Shokohinia Y., Kiani A., Sadrjavadi K., Nowroozi A., Shahlaei M. (2016). Molecular insight into the Grandivitin-matrix metalloproteinase 9 interactions. J. Photochem. Photobiol. B.

[75] Tatevossian R.G., Lawson A.R., Forshew T., Hindley G.F., Ellison D.W., Sheer D. (2010). MAPK pathway activation and the origins of pediatric low-grade astrocytomas. J. Cell. Physiol..

[76] Balmanno K., Cook S. (2009). Tumour cell survival signalling by the ERK1/2 pathway. Cell Death Differ..

[77] Luo J., Manning B.D., Cantley L.C. (2003). Targeting the PI3K-Akt pathway in human cancer: Rationale and promise. Cancer Cell.

[78] Buckner J.C. (2003). Factors influencing survival in high-grade gliomas. Seminars in Oncology.

[79] Chen Y.-H., Hung M.-C., Shyu W.-C. (2012). Role of cancer stem cells in brain tumors. Biomedicine.

[80] Zhang X., Chen T., Zhang J., Mao Q., Li S., Xiong W., Qiu Y., Xie Q., Ge J. (2012). Notch1 promotes glioma cell migration and invasion by stimulating β-catenin and NF-κB signaling via AKT activation. Cancer Sci..

[81] Wilken R., Veena M.S., Wang M.B., Srivatsan E.S. (2011). Curcumin: A review of anti-cancer properties and therapeutic activity in head and neck squamous cell carcinoma. Mol. Cancer.

[82] Jemal A., Siegel R., Ward E., Hao Y., Xu J., Murray T., Thun M.J. (2008). Cancer statistics, 2008. CA Cancer J. Clin..

[83] Parkin D.M., Bray F., Ferlay J., Pisani P. (2005). Global cancer statistics, 2002. CA Cancer J. Clin..

[84] Xu X., Zhang Y., Qu D., Jiang T., Li S. (2011). Osthole induces G2/M arrest and apoptosis in lung cancer A549 cells by modulating PI3K/Akt pathway. J. Exp. Clin. Cancer Res..

[85] Mathisen M.S., Kantarjian H.M., Cortes J., Jabbour E. (2011). Mutant BCR-ABL clones in chronic myeloid leukemia. Haematologica.

[86] Souza P.S., Vasconcelos F.C., De Souza Reis F.R., De Moraes G.N., Maia R.C. (2011). P-glycoprotein and survivin simultaneously regulate vincristine-induced apoptosis in chronic myeloid leukemia cells. Int. J. Oncol..

[87] Echelman D., Feldman S. (2012). Management of cervical precancers: A global perspective. Hematol. Oncol. Clin. N. Am..

[88] Mei L., Chen H., Wei D.M., Fang F., Liu G.J., Xie H.Y., Wang X., Zou J., Han X., Feng D. (2013). Maintenance chemotherapy for ovarian cancer. Curr. Oncol. Rep..

[89] Park J.T., Chen X., Trope C.G., Davidson B., Shih I.-M., Wang T.-L. (2010). Notch3 overexpression is related to the recurrence of ovarian cancer and confers resistance to carboplatin. Am. J. Pathol..

[90] Wells A., Grahovac J., Wheeler S., Ma B., Lauffenburger D. (2013). Targeting tumor cell motility as a strategy against invasion and metastasis. Trends Pharmacol. Sci..

[91] Ahmadi F., Shokoohinia Y., Javaheri S., Azizian H. (2017). Proposed binding mechanism of galbanic acid extracted from *Ferula assa–foetida* to DNA. J. Photochem. Photobiol. B.

[92] Shokoohinia Y., Khajouei S., Ahmadi F., Ghiasvand N., Hosseinzadeh L. (2017). Protective effect of bioactive compounds from *Echinophora cinerea* against cisplatin-induced oxidative stress and apoptosis in the PC12 cell line. Iran. J. Basic Med. Sci..

[93] Shokoohinia Y., Bazargan S., Miraghaee S., Javadirad E., Hosseinzadeh L. (2017). Safety assessment of osthole isolated from *Prangos ferulacea*: Acute and subchronic toxicities and modulation of cytochrome P450. Jundishapur J. Nat. Pharm. Prod..

[94] Davatgaran-Taghipour Y., Masoomzadeh S., Farzaei M.H., Bahramsoltani R., Karimi-Soureh Z., Rahimi R., Abdollahi M. (2017). Polyphenol nanoformulations for cancer therapy: Experimental evidence and clinical perspective. Int. J. Nanomed..

[95] Peiris-Pagès M., Martinez-Outschoorn U.E., Sotgia F., Lisanti M.P. (2015). Metastasis and oxidative stress: Are antioxidants a metabolic driver of progression?. Cell Metabol..

[96] Panieri E., Santoro M.M. (2016). ROS homeostasis and metabolism: A dangerous liaison in cancer cells. Cell Death Dis..

[97] Nelson K.K., Melendez J.A. (2004). Mitochondrial redox control of matrix metallopro-teinases. Free Radic. Biol. Med..

[98] Morry J., Ngamcherdtrakul W., Yantasee W. (2017). Oxidative stress in cancer and fibrosis: Opportunity for therapeutic intervention with antioxidant compounds, enzymes, and nanoparticles. Redox Biol..

